# Enhanced Electrochemical CO_2_ Reduction to Formate on Poly(4-vinylpyridine)-Modified Copper and Gold Electrodes

**DOI:** 10.1021/acsami.2c10452

**Published:** 2022-09-27

**Authors:** Chunmiao Ye, Stefan J. Raaijman, Xiaoting Chen, Marc T. M. Koper

**Affiliations:** Leiden Institute of Chemistry, Leiden University, P.O. Box 9502, 2300 RA Leiden, The Netherlands

**Keywords:** CO_2_RR, poly(4-vinylpyridine), Cu, Au, modification, HCOOH, ATR-SEIRAS, hydrophobic

## Abstract

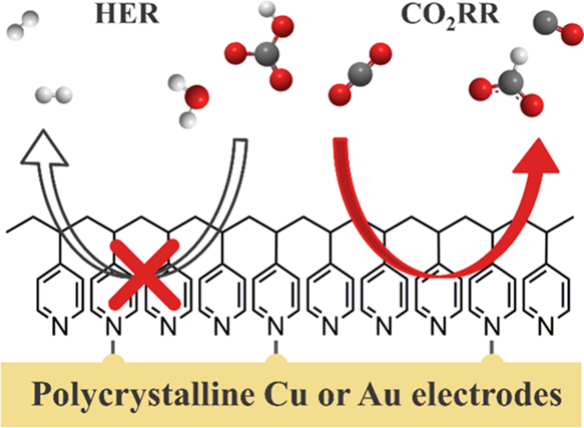

Developing active and selective catalysts that convert CO_2_ into valuable products remains a critical challenge for further application of the electrochemical CO_2_ reduction reaction (CO_2_RR). Catalytic tuning with organic additives/films has emerged as a promising strategy to tune CO_2_RR activity and selectivity. Herein, we report a facile method to significantly change CO_2_RR selectivity and activity of copper and gold electrodes. We found improved selectivity toward HCOOH at low overpotentials on both polycrystalline Cu and Au electrodes after chemical modification with a poly(4-vinylpyridine) (P4VP) layer. In situ attenuated total reflection surface-enhanced infrared reflection-adsorption spectroscopy and contact angle measurements indicate that the hydrophobic nature of the P4VP layer limits mass transport of HCO_3_^–^ and H_2_O, whereas it has little influence on CO_2_ mass transport. Moreover, the early onset of HCOOH formation and the enhanced formation of HCOOH over CO suggest that P4VP modification promotes a surface hydride mechanism for HCOOH formation on both electrodes.

## Introduction

The electrochemical CO_2_ reduction reaction (CO_2_RR) provides a promising route to utilize carbon feedstock and store renewable electrical energy. Various products, such as formic acid, carbon monoxide, hydrocarbons, and alcohols, can be obtained via the CO_2_RR in aqueous media. Extensive experimental and theoretical work has been dedicated to investigate the CO_2_RR process to different products.^1−4^ The initial two-electron transfer products formed during the CO_2_RR in aqueous media are formic acid (HCOOH) and carbon monoxide (CO), with subsequent further reduced products commonly agreed upon as resulting from CO reduction (mainly on copper (Cu) catalysts).^5^ Because of the high ratio of molecular weight per electron transfer, formic acid has been considered one of the most economically interesting products.^6,7^ However, HCOOH-selective catalysts such as Sn, Pb, and Pd require high overpotentials (>0.8 V) or suffer from low stability,^8−10^ which limits their application. Therefore, discovering the factors that govern the selectivity of CO_2_RR to HCOOH could open up the possibility of selective HCOOH synthesis from CO_2_RR with higher efficiency.

Despite extensive efforts of the scientific community, achieving high selectivity at low overpotentials remains a significant challenge for the CO_2_RR.^11−14^ One strategy to influence product selectivity is catalyst modification with organic additives such as poly(4-vinyl pyridine) (P4VP),^15^ N-substituted pyridinium,^16^ poly(acrylamide) (PAM),^17^ and *N*,*N*′-ethylene-phenanthrolinium dibromide.^18^ Coating polycrystalline Cu electrodes with P4VP yields improved formic acid selectivity with maximum faradaic efficiency (FE) of ca. 40%.^15^ Polycrystalline Cu electrodes modified with N-substituted pyridinium additives instead produce C_2_ and C_3_ products with total FE of ca. 70–80% (although these electrodes also show enhanced formic acid formation at low overpotentials).^16^ Cu surface modification by polyaniline results in similar behavior, improving selectivity toward C_2+_ hydrocarbons to ca. 80%.^19^ There are two main considerations explaining how additives affect the activity and selectivity of CO_2_RR catalysts: (i) by influencing the catalytic activity (by stabilizing/destabilizing reaction intermediates) and/or (ii) by changing the local concentration of interfacial species involved in the reaction.^20,21^ To illustrate, a higher CO coverage on polyaniline coated polycrystalline Cu catalysts,^19^ an increased local pH for polycrystalline Cu electrodes modified with N-substituted pyridinium additives,^16^ and enhanced stabilization of the CO dimer on PAM modified Cu polycrystalline catalysts^17^ have been speculated to increase the selectivity toward C_2+_ hydrocarbons, whereas the unfavorable H_2_O dissociation and limited mass transport of proton donors (H_2_O and HCO_3_^–^) have been proposed to lead to the suppression of the hydrogen evolution reaction (HER) on alkanethiol-modified Cu mesh electrodes^22^ and cetalkonium chloride-modified polycrystalline Sn eletrodes.^23^ Furthermore, recent computational studies have demonstrated a relation between changes in hydrophobicity resulting from functionalization of a Cu surface with organic molecules and the tendency to form surface hydrides.^24^ In the latter work, it was proposed that hydrophilic interfaces promote the formation of surface hydrides, which enhance the formation of formic acid, while hydrophobic interfaces favor CO formation instead.^24^ These proposed explanations on the role of additives were mainly focused on multicarbon products. More experimental evidence to elucidate the enhanced HCOOH formation is still highly desirable.

To investigate to what extent organic additives affect CO_2_RR activity and selectivity, we studied the CO_2_RR on polycrystalline Cu (poly Cu) and Au (poly Au) electrodes with and without P4VP layer coating, focusing on changes in the catalytic activity and product distribution. In agreement with the literature,^15^ we observe an increase in selectivity of CO_2_RR to HCOOH on a P4VP-modified polycrystalline Cu (P4VP-modified Cu) electrode. Interestingly, this same effect is also observed on a P4VP-modified polycrystalline Au (P4VP-modified Au) electrode, even though pristine Au is a highly selective catalyst for reducing CO_2_ to CO.^30,31^ Our results suggest that surface modification with a P4VP layer results in enhanced selectivity toward HCOOH during the CO_2_RR, regardless of the nature of electrocatalysts. To better understand this behavior, we employed in situ attenuated total reflection surface-enhanced infrared reflection-adsorption spectroscopy (ATR-SEIRAS) to investigate the interfacial reaction species during the CO_2_RR on P4VP-modified electrodes. We show that, apart from the interaction between the P4VP layer and the metal catalysts influencing catalysis, organic layer-induced limitations in mass transport of H_2_O and HCO_3_^–^ result in a local environment rich in CO_2_, which thereby increases the CO_2_RR rate whilst suppressing the HER. Finally, the selective enhancement of HCOOH formation over CO at low overpotentials suggests a surface hydride pathway to HCOOH. Our work thereby offers a more comprehensive understanding of the role of the P4VP layer in tuning CO_2_RR activity and selectivity.

## Results and Discussion

**Figure 1:**
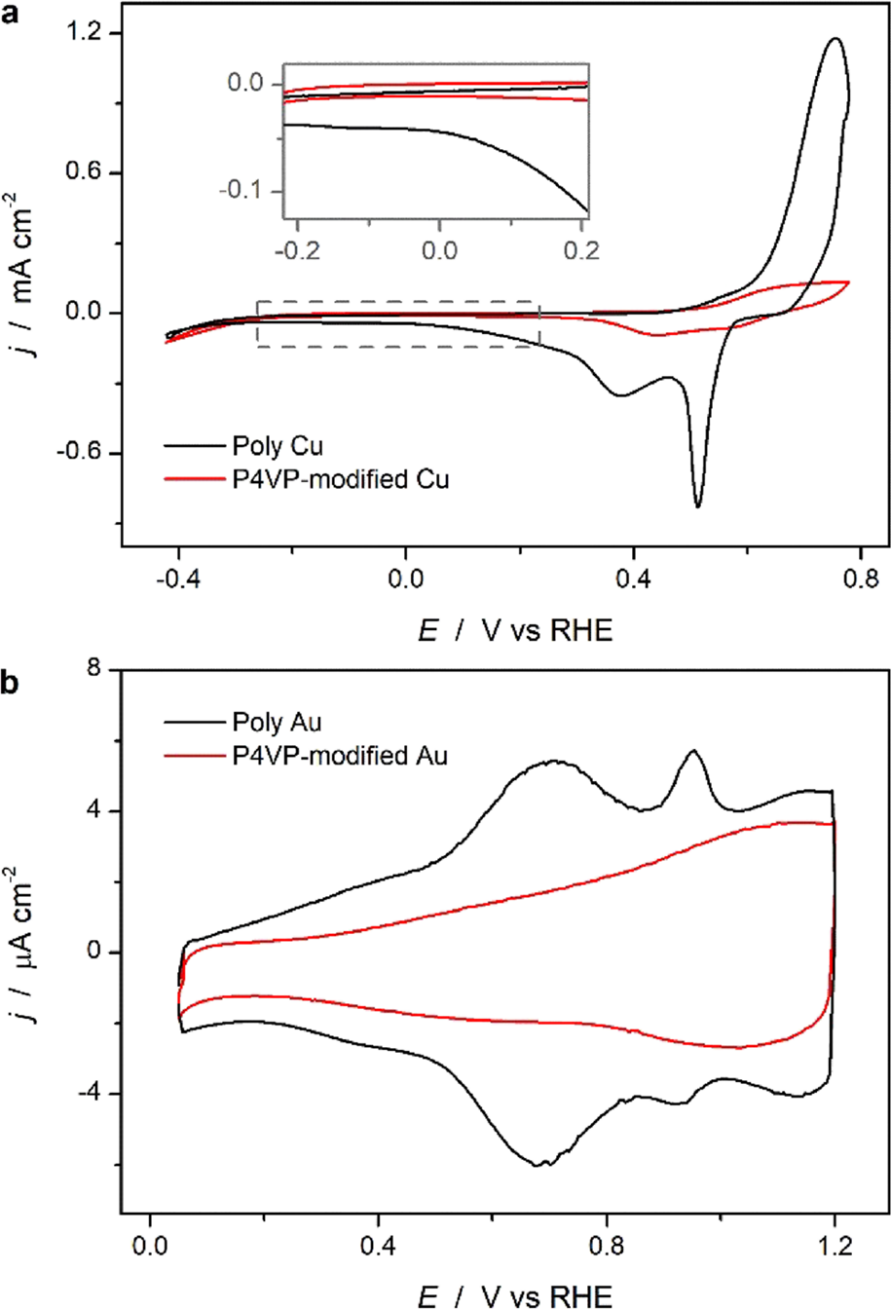
Cyclic voltammograms of (a) poly Cu and (b) poly Au with (red) and without (black) a P4VP layer, measured at 50 mV/s in CO_2_ saturated 0.1 M KHCO_3_ solution.

Cyclic voltammetry in aqueous CO_2_-saturated 0.1 M KHCO_3_ was used to characterize the initial state of the electrodes. Figure 1a shows the cyclic voltammograms of the unmodified poly Cu (black curve) and the P4VP-modified Cu electrodes (red curve), respectively. The poly Cu electrode shows a cyclic voltammogram similar to that reported before,^32,33^ characterized by peaks corresponding to surface oxidation/reduction. The oxidation peak during the positive-going scan is the result of Cu oxide formation (Cu^0^ → CuO), while the reduction peaks are associated with the reverse reaction, being CuO → Cu_2_O and Cu_2_O → Cu^0^, respectively, when scanning from positive to negative.^34,35^ Much decreased double layer charging current (−0.2 to 0.2 V vs RHE) and oxidation current (0.5–0.8 V vs RHE) are observed on the P4VP-modified Cu electrode, implying that fewer Cu sites are electrochemically accessible because of the presence of the P4VP layer.

Gold was characterized similarly (Figure 1b), where the reversible peaks at 0.7 and 0.95 V vs RHE in this case correspond to the electrochemical lifting of the Au (110) and Au (111) surface reconstructions. Although the peaks associated with the lifting of the surface reconstruction of gold have been associated with sulfate adsorption,^36,37^ they were also observed in 0.1 M KHCO_3_ solutions in a previous work reported by our group.^38^ With the presence of a P4VP layer, these reconstruction peaks diminish greatly, leaving a broad double layer current, which confirms the decreased permeability of the electrolyte to the reaction interface.

In addition to cyclic voltammetry, atomic force microscopy (AFM) was used to characterize the morphology of the electrodes, as depicted in Figure S1. Figure S1a,c represents poly Cu and poly Au surfaces, respectively, with both electrodes showing typical metallic polycrystalline surfaces with grain boundaries. After modification with the P4VP layer, for P4VP-modified Cu (Figure S1b) and Au electrodes (Figure S1d), respectively, the morphologies of the surfaces become smoother compared to the uncoated surfaces. This indicates that the P4VP layer fully covers the electrode surfaces, yielding a significant reduction in surface roughness. Additionally, dark spots can be observed in Figure S1b, d, representing small holes in P4VP film, which confirms previous reports of a P4VP layer adopting a mesoporous structure.^39^

**Figure 2:**
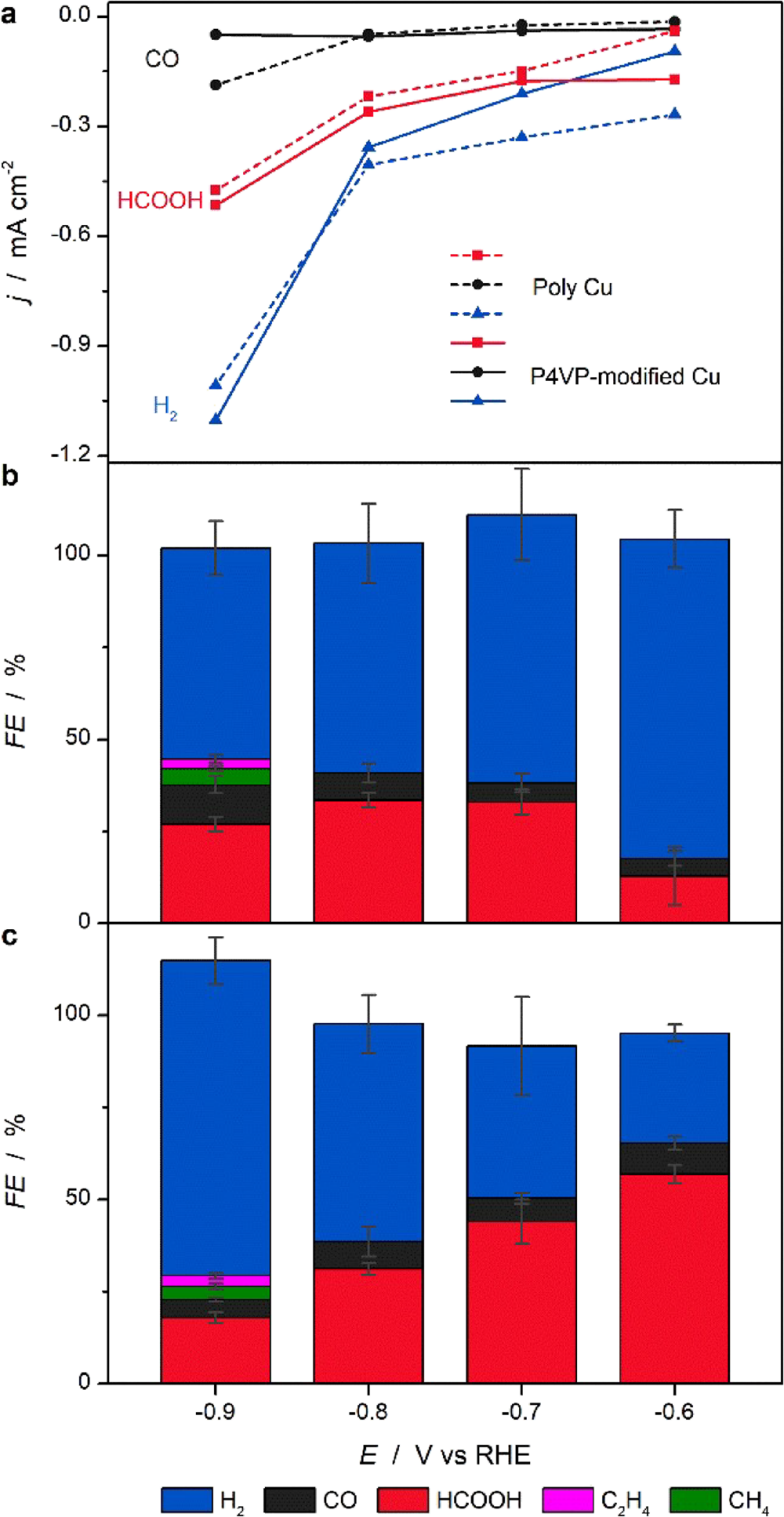
CO_2_RR performance in CO_2_ saturated 0.1 M KHCO_3_. (a) Partial current density of H_2_, HCOOH, and CO as a function of potential on unmodified Cu (dashed line) and P4VP-modified Cu (solid line), with the FEs for observed products on (b) poly Cu and (c) P4VP-modified Cu. Error bars are standard deviations based on three measurements.

Figure 2 depicts the effects on CO_2_RR performance after chemical modifications of a polycrystalline Cu surface with a P4VP layer. The partial current densities of the dominant products (H_2_, CO, and HCOOH) are shown in Figure 2a, with the dashed and solid lines representing unmodified and P4VP-modified Cu electrodes, respectively. Associated FEs (including observed minority products) are depicted in Figure 2b, c, for the unmodified and P4VP-modified Cu electrodes, respectively. Even though other products (ethanol, *n*-propanol etc.) have been reported as possible products,^1^ they were not detected under our working conditions. The total current density of CO_2_RR-related products on P4VP-modified Cu is higher than that on unmodified Cu at low overpotentials. This is especially clear at −0.6 V vs RHE, where the combined partial current density of CO_2_RR-related products on the P4VP-modified Cu electrode is almost four times higher than that on unmodified Cu. Additionally, at this potential, the current density for H_2_ production on the P4VP-modified Cu electrode is a factor of three lower. These results demonstrate that the presence of the P4VP layer promotes the reduction of CO_2_ and simultaneously suppresses the HER, especially at low overpotentials. Besides enhancing the total activity of the CO_2_RR, product selectivity is also changed. FEs for HCOOH, CO, and H_2_ are 13, 5, and 87%, respectively, for unmodified Cu at −0.6 V vs RHE, whilst on P4VP-modified Cu, the FEs are 57, 8, and 33%, respectively, leading to an enhancement factor of 4.4 for HCOOH and 1.6 for CO. The partial current density and FE for CO_2_RR products (HCOOH and CO) on P4VP-modified Cu remains higher than those for the unmodified electrode at −0.7 V vs RHE. However, at more negative potentials, especially at −0.9 V vs RHE, the partial current density of CO on the unmodified Cu electrode increases, while it remains at the same level as less negative potential on the P4VP-modified Cu electrode. Correspondingly, the FEs of CO, CH_4_, and C_2_H_4_ on P4VP-modified Cu are lower than those of the unmodified Cu electrode. Our data therefore suggest that the P4VP layer enhances HCOOH formation and increases the selectivity of HCOOH at low overpotentials, while it has negative effect on the selectivity of CH_4_ and C_2_H_4_ at high overpotentials.

**Figure 3:**
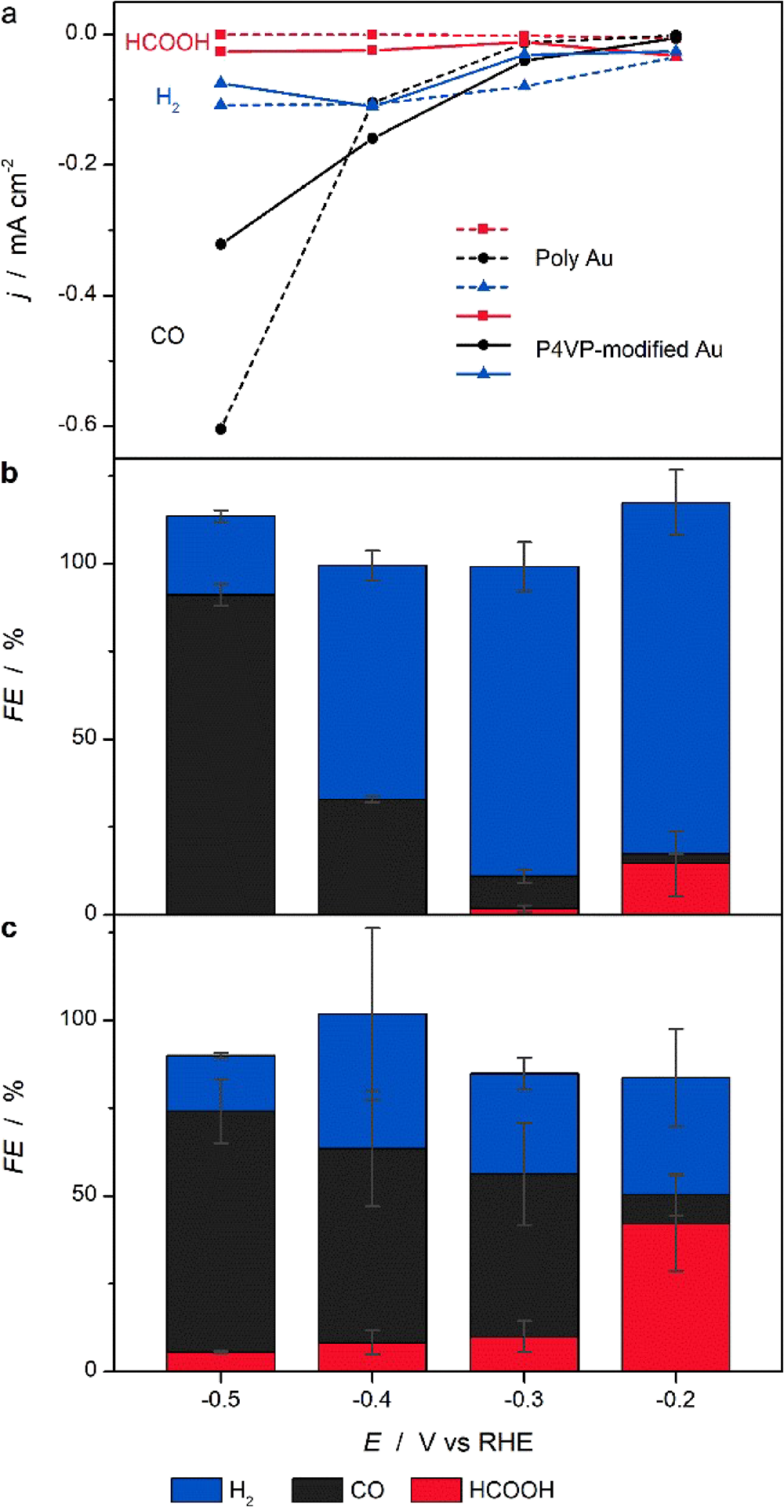
CO_2_RR performance in CO_2_-saturated 0.1 M KHCO_3_. (a) Partial current density of H_2_, HCOOH, and CO as a function of potential on poly Au (dashed line) and P4VP-modified Au (solid line), with the FEs for observed products on (b) poly Au and (c) P4VP-modified polycrystalline Au shown as bars. Errors bars are standard deviations based on three measurements.

To gain additional insights into the mechanism underlying the observed P4VP effects, we continue to investigate the CO_2_RR on unmodified and P4VP-modified poly Au electrodes. Figure 3 shows CO_2_RR performance of unmodified and P4VP-modified Au electrodes in CO_2_-saturated 0.1 M KHCO_3_. It can be seen in Figure 3a (dashed line) and 3b that, at low overpotentials, unmodified poly Au predominantly forms hydrogen with only minor amounts of HCOOH and CO, with the former having a relatively higher FE. At increasingly negative potentials, CO_2_ reduction gradually takes over, and CO becomes the dominant product whilst HCOOH disappears entirely. In contrast, HCOOH formation is observed on P4VP-modified Au in the entire investigated potential region, as shown in Figure 3a (solid line) and Figure 3c. Notably, HCOOH is the dominant product at −0.2 V vs RHE with a FE of 42%, although the overall rate is low.

To exclude the possible catalytic effect of the P4VP layer itself, we performed CO_2_ reduction on unmodified pyrolytic graphite and P4VP-modified pyrolytic graphite electrodes (with the pyrolytic graphite electrode being chosen for its poor CO_2_RR performance). Measured cyclic voltammograms and the obtained total current densities at −0.6 V vs RHE on the pyrolytic graphite and P4VP-modified pyrolytic graphite electrodes are shown in Figure S2. No CO_2_RR products were detected. Therefore, the P4VP layer itself is catalytically inert. This result confirms that the effect of P4VP layer on the activity and selectivity of the CO_2_RR results from the interaction of the P4VP layer and metallic active sites (poly Cu and Au).

**Figure 4:**
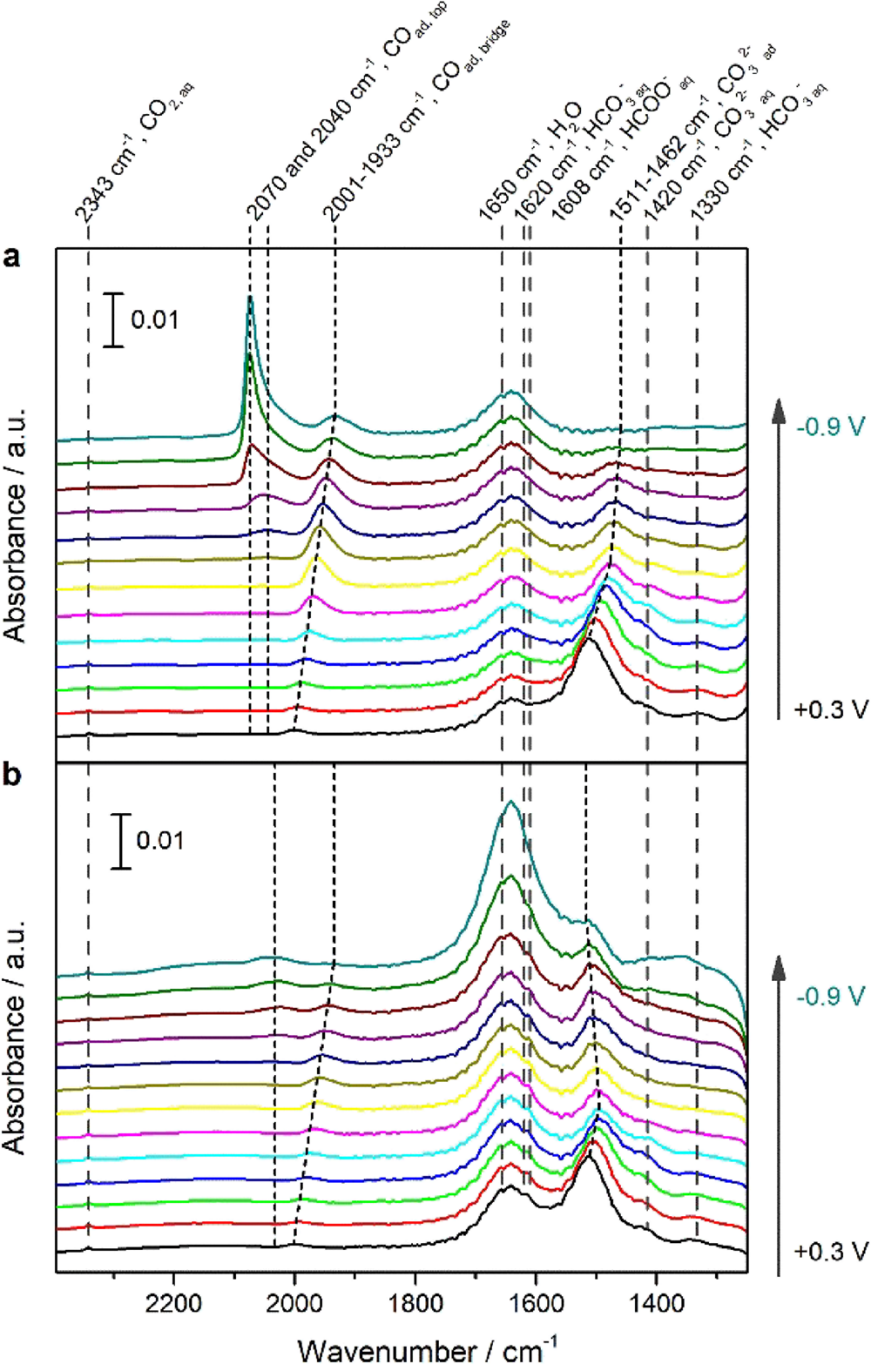
ATR-SEIRA spectra of the CO_2_RR on (a) poly Cu and (b) P4VP-modified Cu during linear sweep voltammetry at 1 mV/s from 0.2 to −0.9 V vs RHE in CO_2_ saturated 0.1 M KHCO_3_. The background spectrum was taken at OCP in H_2_O before experiments. The potential interval between spectra is 0.1 V.

In an attempt to unravel the origin of the improved CO_2_RR performance, we further employed in situ ATR-SEIRAS to study the surface adsorbates, as well as the interfacial electrolyte species. Figure 4 shows the in situ ATR-SEIRA spectra recorded on unmodified Cu (Figure 4a) and P4VP-modified Cu (Figure 4b) during linear sweep voltammetry (LSV) experiment, scanning from +0.3 to −0.9 V vs RHE at a scan rate of 1 mV/s in CO_2_-saturated 0.1 M KHCO_3_, with the background spectra taken at OCP in Milli-Q water. Besides the H_2_O bending peak at 1650 cm^–1^,^25,29,40,41^ various bands related to surface adsorbates and electrolyte species are observed in the ATR-SEIRA spectra, with an overview of the different bands pertinent to this work being provided in Table S1. It can be seen in Figure 4a that, on a polycrystalline copper surface, the dominant bands in the ATR spectrum at +0.3 V vs RHE are located at 1640 and 1511 cm^–1^, and can be attributed to a combination of the H_2_O bending band (1650 cm^–1^) and the asymmetrical stretching of HCO_3_^–^ in solution (1620 cm^–1^) for the former,^25,26,29,41^ with the latter being indicative of adsorbed CO_3_^2–^.^28,29,42^ Additional minor bands are located at 2343, 2001, 1420, and 1330 cm^–1^, which correspond to solution phase (dissolved) CO_2_,^43^ bridge-bound CO (CO_ad,bridge_), and solution-phase CO_3_^2–^ and HCO_3_^–^.^26,27,29,42,44^ When the potential is scanned to more negative potentials, the band associated with adsorbed CO_3_^2–^ red-shifts from 1511 cm^–1^ at +0.3 V vs RHE to 1462 cm^–1^ at −0.9 V vs RHE as a result of the Stark tuning effect, with the intensity of this band gradually decreasing as CO_3_^2–^ starts to desorb due to the negatively charged interface at more negative potentials.^28,29,42^ The band at 2001 cm^–1^ (related to CO_ad,bridge_) is similarly found to redshift when the potential is scanned to more negative values whilst simultaneously increasing in intensity, whereas the band at 2343 cm^–1^ diminishes due to solution-phase CO_2_ being depleted by the CO_2_RR at increasingly negative potentials. As for the bands related to solution-phase CO_3_^2–^ and HCO_3_^–^, their intensity is too weak to make meaningful observations and we will therefore not further discuss. Besides these bands, a number of additional bands starts to appear during the negative-going scan. Although CO_ad,bridge_ is visible already at +0.3 V vs RHE, we know from experiments conducted at higher potential that this band starts to increase from −0.1 V vs RHE. Therefore, we believe that CO_ad,bridge_ at the onset of LSV is an artifact from the initial electrochemical preparation of the surface, with the actual onset of this band being at −0.1 V vs RHE. When the potential is scanned further negative, a bread peak at 2050 cm^–1^ starts to appear from −0.5 V vs RHE, ascribed to top-bound CO (CO_ad,top_) on Cu (100) terrace sites.^44^ The appearance of this peak is accompanied by the formation of a sharp band at 2070 cm^–1^, which is assigned to CO_ad,top_ on Cu (100) and (111) step sites.^45,46^ The latter band becomes increasingly dominant as the potential is scanned further toward −0.9 V vs RHE. Overall, on the poly Cu surface, the adsorbed species as well as solution species change as a function of the applied potential, the main adsorbates being CO_3_^2–^_ad_ at low overpotential and adsorbed CO at a more negative potential.

A new working electrode was prepared for ATR-SEIRAS experiments on P4VP-modified Cu. To ensure system integrity and surface reproducibility, the copper surface was characterized with ATR-SEIRAS prior to adding a P4VP layer, although the LSV was halted at −0.5 V vs RHE to ensure stability of the surface. As shown in Figure S3, the presence of CO_ad,top_ and CO_ad,bridge_ confirms the reproducibility of ATR-SEIRA spectra on the poly Cu surface. The in situ ATR-SEIRA spectra obtained on a P4VP-modified Cu electrode are depicted in Figure 4b. Although the P4VP layer seems less stable at the end of LSV (band related to H_2_O and HCO_3_^–^ at 1620 cm^–1^ starts to increase at −0.8 and – 0.9 V vs RHE), the bands corresponding to different interfacial species are located at the same positions during the CO_2_RR. However, after P4VP modification, an additional band at 1608 cm^–1^ is observed, which is assigned to the asymmetric stretching of solution-phase HCOO^–^.^29^ Contrary to unmodified Cu, at more negative potentials, the band related to CO_ad_,_top_ on (100)/(111) step sites (2070 cm^–1^) is barely present on P4VP-modified Cu, while the bands corresponding to adsorbed CO_ad,top_ and CO_ad,bridge_ on (100) terraces (2040 and 2001 cm^–1^, respectively) exhibit reduced intensity. With higher CO production (see partial current density, Figure 2a) on P4VP-modified Cu between −0.6 and −0.8 V vs RHE, these differences in the CO adsorption band indicate fewer active sites on P4VP-modified Cu, which suggests that the P4VP layer coordinates with Cu active sites, especially with step sites. Furthermore, these reduced CO adsorption bands could also explain lower selectivity of CH_4_ and C_2_H_4_ on the P4VP-modified Cu electrode. Overall, the discussed results reveal the effect of the P4VP layer on adsorbed CO, suggesting an interaction between the P4VP layer and metallic poly Cu surface. However, the ATR-SEIRAS results do not give a clear indication of the effect of the P4VP layer on interfacial solution species due to the low band intensity.

**Figure 5:**
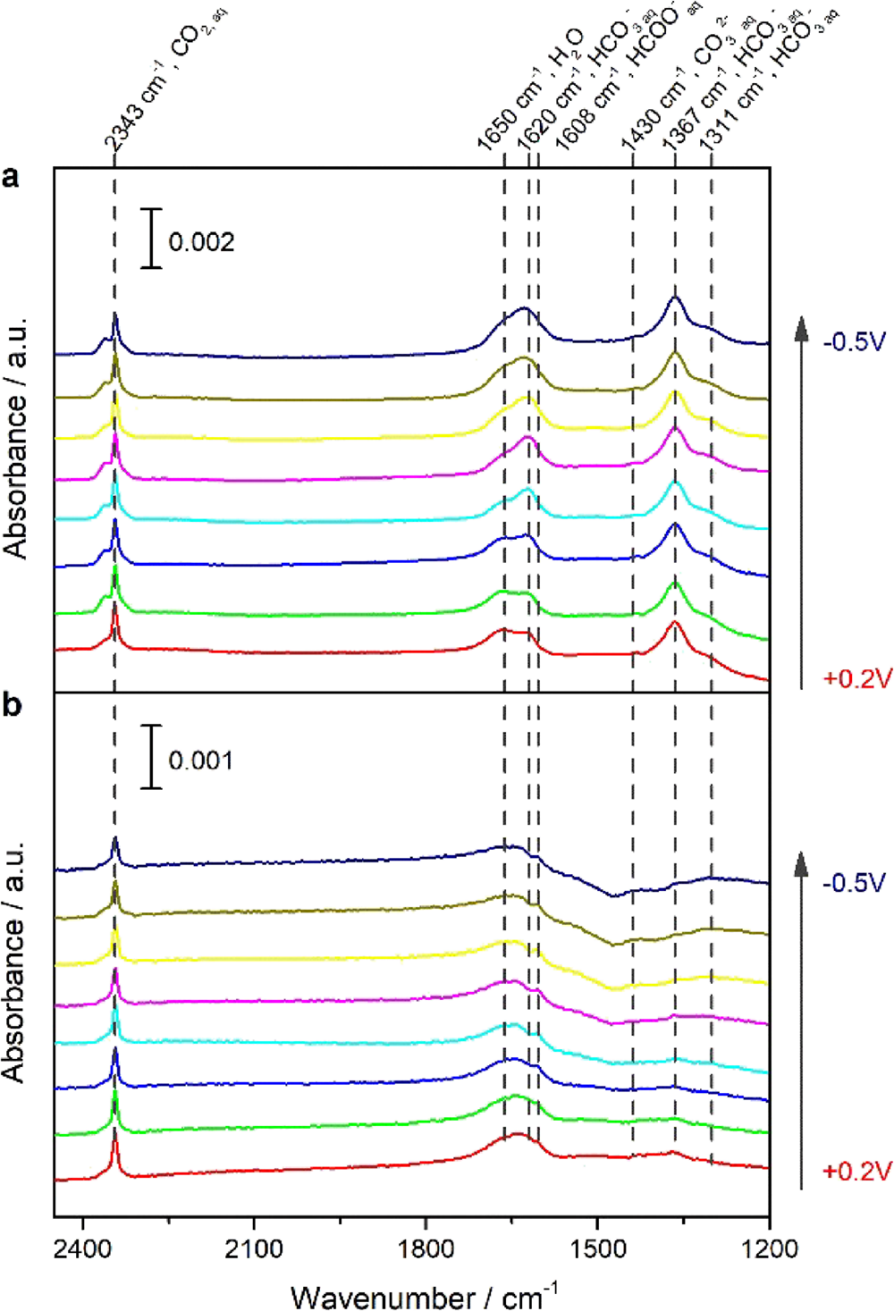
ATR-SEIRA spectra of the CO_2_RR on (a) poly Au and (b) P4VP-modified Au during linear sweep voltammetry at 1 mV/s from 0.2 to −0.5 V vs RHE in CO_2_-saturated 0.1 M KHCO_3_. The background spectrum was taken at OCP in pure H_2_O prior to ATR-SEIRAS experiments. The potential interval between spectra is 0.1 V.

**Figure 6:**
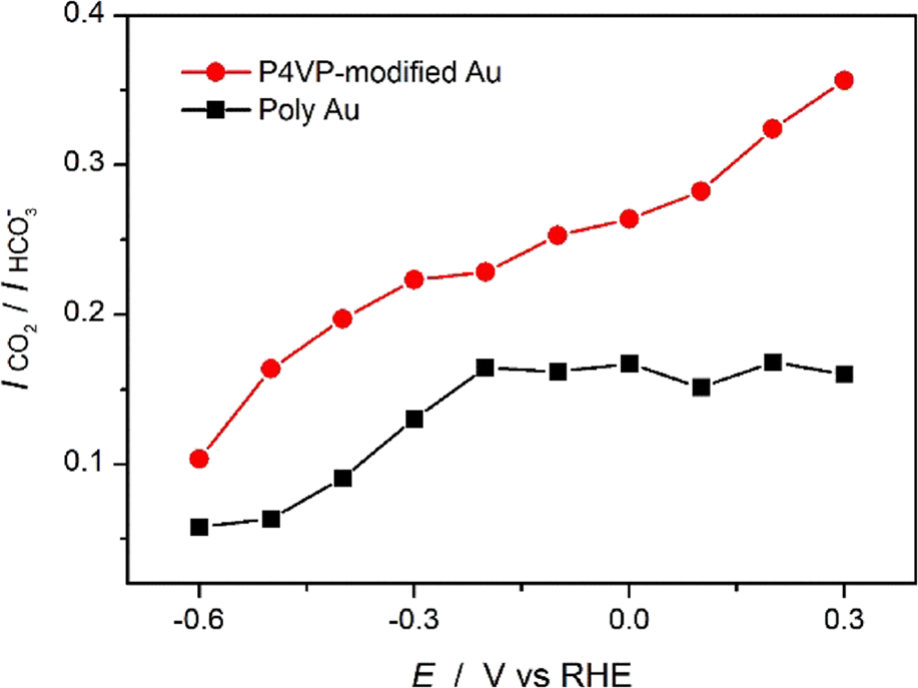
Potential dependency of *I*_CO2_/*I*_HCO3_^–^ on poly Au (black) and P4VP-modified Au (red), calculated from ATR-SEIRA spectra obtained during LSV at 1 mV/s in CO_2_ saturated 0.1 M KDCO_3_ in D_2_O. The background spectrum was taken at OCP in D_2_O before experiments.

In the previous literature,^25,26^ ATR-SEIRAS experiments were applied to determine local pH via the ratio of carbon dioxide and bicarbonate vibrational bands on polycrystalline Au electrodes. To gain further insight into the effect of the modification by P4VP on the near-electrode solution-phase species, we performed in situ ATR-SEIRAS on unmodified Au and P4VP-modified Au electrodes. Figure 5a, b shows the recorded ATR-SEIRA spectra during LSV at 1 mV/s in CO_2_ saturated 0.1 M KHCO_3_ for unmodified and P4VP-modified Au, respectively. Although adsorbed CO bands were observed around 2000 cm^–1^ on Au electrodes in previous work,^38,47,48^ they were not observed under our working conditions. We observe only bands related to H_2_O and solution-phase species; CO_2_, HCO_3_^–^, CO_3_^2–^. Additionally, on P4VP-modified Au, there is a very weak feature at 1608 cm^–1^, which may be assigned to solution HCOO^–^.^29^ However, the band attributed to electrolyte HCO_3_^–^ has a much lower relative intensity on the P4VP-modified Au surface, suggesting different local environments after adding the P4VP layer. Instead of looking at absolute band intensity, the ratio of the integrals of the bands related to dissolved CO_2_ and solution HCO_3_^–^ (2343 and 1620 cm^–1^, respectively; *I*_CO2_/*I*_HCO3_^–^) was calculated as an indicator of local environment. To avoid convolution with the H_2_O bending band, *I*_CO2_/*I*_HCO3_^–^ was determined from in situ ATR-SEIRA spectra taken in D_2_O, as the D_2_O bending band is shifted to 1208 cm^–1^. Figure S4 shows the ATR-SEIRA spectra, obtained on unmodified Au and P4VP-modified Au electrodes in CO_2_ saturated 0.05 M K_2_CO_3_ in D_2_O. The calculated *I*_CO2_/*I*_HCO3_^–^ ratio as a function of applied potential for both surfaces is shown in Figure 6. On unmodified Au electrodes, the obtained *I*_CO2_/*I*_HCO3_^–^ agrees with literature reports.^25,26^ Specifically, in the potential region of 0.3 to −0.1 V vs RHE, where no HER nor CO_2_RR occurs, *I*_CO2_/*I*_HCO3_^–^ remains constant; when the negative-going scan enters the region of −0.2 to −0.4 V vs RHE, where HER and CO_2_RR start, *I*_CO2_/*I*_HCO3_^–^ keeps decreasing because of the CO_2_ consumption and HCO_3_^–^ formation, as a consequence of the reaction between CO_2_ and OH^–^; finally, from −0.5 V vs RHE onward, *I*_CO2_/*I*_HCO3_^–^ reaches the lowest point, where CO_2_ is almost depleted and HCO_3_^–^ starts to be consumed by the excess OH^–^, and as a result, CO_3_^2–^ finally becomes the dominant solution species near the electrode. On the other hand, on the P4VP-modified Au, the obtained *I*_CO2_/*I*_HCO3_^–^ (Figure 6, red line) is always higher than that on the unmodified Au and keeps decreasing with the negative-going scan. The higher value of *I*_CO2_/*I*_HCO3_^–^ in the potential region between 0.3 and – 0.1 V vs RHE indicates the limited mass transport of HCO_3_^–^ (compared with CO_2_) from the bulk electrolyte to the P4VP–Cu interface. Moreover, the decreasing *I*_CO2_/*I*_HCO3_^–^ in this potential region (where no reaction occurs) suggests slow HCO_3_^–^ diffusion from the bulk electrolyte to the P4VP–Cu interface. Therefore, we propose that this water-insoluble P4VP layer not only limits the mass transport of H_2_O but also limits the mass transport of HCO_3_^–^. Both species are proton donor of HER during the CO_2_RR, thereby lowering HER activity. As a result, less OH^–^ formation is expected due to the limited HER, and hence, less CO_2_ is consumed by homogeneous reaction (with OH^–^) during the CO_2_RR, which could explain the higher *I*_CO2_/*I*_HCO3_^–^ on the P4VP-modified surface at a more negative potential. Overall, we show the *I*_CO2_/*I*_HCO3_^–^ as an indicator of the local environment, which is the CO_2_/HCO_3_ equilibrium resulting from reactions (both HER and CO2RR) at the interface as well as the mass transport from the bulk electrolyte to the interface. We have assigned the inhibited HER on P4VP-modified Cu and Au electrodes to the limit transport of H_2_O and HCO_3_^–^, however, the CO_2_RR products distribution still depends on the nature of the catalysts and the applied potential. Therefore, it is not possible to directly correlate *I*_CO2_/*I*_HCO3_^–^ ratios with CO or HCOOH yield.

As a final consideration, we investigated surface hydrophobicity, as an indicator of surface and H_2_O interaction, via contact angle measurements. Figure S5 shows the contact angles between a water droplet and our investigated surfaces. Unmodified Au shows the most hydrophobic surface with a contact angle of 91°. Upon modification with a P4VP layer, both Cu and Au electrodes shows the same contact angle (57°), which is more hydrophilic than poly Au. Surprisingly, unmodified poly Cu exhibits the most hydrophilic surface (17°), which is probably due to the presence of a CuOx layer when the copper surface is exposed to air. However, CV preparation employed in this work prior to the CO_2_RR experiments is expected to remove this CuOx layer, and therefore, a more hydrophobic surface of metallic poly Cu is expected under CO_2_RR conditions. Overall, the contact angle measurements show different interactions between H_2_O and the employed working electrodes. It has been suggested previously that hydrophilic additives improve HCOOH selectivity during the CO_2_RR, by influencing the formation of surface hydrides.^24^

## Conclusions

In this work, we investigated the effect of chemical modifications via the addition of a P4VP layer on CO_2_RR of poly Cu and Au electrodes. We have shown that the presence of P4VP layer hinders the HER while it enhances the CO_2_RR, especially HCOOH formation, on both electrodes. Less CO-adsorption bands on P4VP-modified Cu and higher *I*_CO2_/*I*_HCO3_^–^ ratios on P4VP-modified Au are observed from in situ ATR-SEIRAS experiments, compared to unmodified electrodes. This indicates inhibited mass transport of H_2_O and HCO_3_^–^ from the bulk to the catalytically active sites with the presence of a P4VP layer, whilst also suggesting coordination between the P4VP layer and Cu sites. In addition, contact angle measurement shows that the P4VP modification influences the hydrophilicity of the surface, which influences surface hydride formation,^24^ resulting in enhanced and preferential HCOOH formation at low overpotentials (with respect to CO). We believe that this is an important experimental finding highlighting that the electrolyte side of the catalyst is very important in steering selectivity, so much so that even a catalyst such as Au can produce substantial amounts of HCOOH. Our work offers further understanding of enhanced CO_2_RR activity and selectivity on P4VP layer modified electrodes and confirms that functionalization by tailored additives is a promising strategy for developing selective catalysts.

## Experimental Section

### Cleaning

Milli-Q water (resistivity >18.2 MΩ·cm, TOC < 5 ppb) was used for all experiments in this work. Prior to each experiment, all cell compartments were cleaned by soaking in an aqueous solution of 1 g·L^–1^ KMnO_4_ (Fluka, ACS reagent) and 0.5 M H_2_SO_4_ (Fluka, ACS reagent) overnight. The solution was subsequently drained, and the cell compartments were rinsed with a dilute piranha solution (1:3 v/v of H_2_O_2_ (Merck, Emprove exp)/H_2_SO_4_) to remove residual KMnO_4_ and MnOx. Afterward, the cell compartments were cleaned by repetitively rinsing and boiling with Milli-Q water to remove all inorganic contaminants.

### Electrode Preparation

A polycrystalline Cu disk electrode (Mateck, 99.995%), a polycrystalline Au disk electrode (Mateck, 99.95%) and a pyrolytic graphite disk electrode (PY001009, Graphite Store) were used as working electrodes. Prior to each experiment, working electrodes were mechanically polished with a diamond suspension (MetaDi, 0.5 μm, Buehler) on a microcloth (Buehler), followed by rinsing with Milli-Q water to remove residual diamond suspension. Afterward, polycrystalline copper and polycrystalline gold electrodes were electropolished as per the following procedures. Polycrystalline copper electrodes were electropolished in a 10:5:2 solution of H_3_PO_4_ (Merck, 85%): H_2_O: H_2_SO_4_ (Fluka, ACS Reagent) at +3 V vs a graphite electrode for 30 s. Polycrystalline Au electrodes were first oxidized in 0.1 M H_2_SO_4_ at +10 V vs the graphite electrode for 20 s and then dipped into 6 M HCl (Merck, ACS reagent, 37%) for 30 s to remove the oxide layer. The obtained electropolished electrodes were further rinsed with Milli-Q water and dried under compressed air flow. P4VP-modified electrodes were obtained by dropcasting 10 μL of 100 mg mL^–1^ P4VP (Aldrich, *M*_w_ ∼ 60,000) in dichloromethane (Actu-all chemicals) solution on dried polycrystalline Cu and Au disk electrodes and drying the electrodes in air for 30 min until all dichloromethane had evaporated.

### Surface Characterization

All AFM imaging was carried out in air with a JPK NanoWizard 3. A SNL (Bruker, resonance frequency: 65 kHz, spring constant: 0.35 N/m) tip was used. The AFM scan rate was 1 Hz, and the images were taken in tapping mode. All the contact angle measurements were carried out with a contact angle goniometer (Ramé-hart, model 250): 3 μL of Milli-Q H_2_O was dropped onto the electrode, and the images were taken within 5 s after H_2_O was dropped on the electrodes. The contact angle measurements were performed three times to confirm the trend; all electrodes were freshly prepared each time.

### Electrochemistry

All electrochemical experiments were carried out in a H-type electrochemical cell equipped with three electrodes. The cathode and anode were separated by a Nafion 117 membrane (Aldrich, thickness: 0.0006 inch). A dimensionally stable anode (Magneto Special Anodes) was used as a counter electrode, and a leakless Ag/AgCl (EDAQ) was used as a reference electrode. All reported potentials were converted to the reversible hydrogen electrode (RHE) scale. All working potentials were controlled with a Bio-logic SP-300 Potentiostat under room temperature. The working electrolyte was an aqueous 0.1 M KHCO_3_ solution, made from Milli-Q water and KHCO_3_ (Fluka, ACS reagent). Before every CO_2_ reduction experiment, CO_2_ (Linde, 4.5) was bubbled through the electrolyte for 30 min to obtain a CO_2_ saturated solution. Blank cyclic voltammograms were first taken to characterize the surface and to stabilize the initial state of the electrode. The Ohmic resistance was evaluated by electrochemical impedance spectroscopy (EIS) at 0.1 V vs RHE, and 85% Ohmic drop compensation was applied to all subsequent experiments. Electrolysis was performed at fixed potentials for 30 min, constantly purging the electrolyte at 8 mL/min CO_2_ for a stable pH and continuous CO_2_ supply. Gas and liquid samples were taken every 10 min. After all experiments, P4VP layer remains intact on both poly Cu and Au electrodes under our working conditions, as evidenced by the cyclic voltammograms of P4VP-modified electrodes after CO_2_RR in Figure S6.

The gas products from CO_2_ reduction were analyzed using a GC-2010 plus system (Shimadzu). The GC system was equipped with two columns. A RTX-1 column (Restek) connected to a FID was used to separate and detect hydrocarbons (CH_4_, C_2_H_4_), and a ShinCarbon ST micropacked 80/100 column (Restek) connected to a TCD was used for H_2_ and CO. Liquid products were analyzed via HPLC with an Aminex HPX-87H column (BioRad) equipped with an RID detector (Shimadzu). The detection limit for H_2_, CO and HCOOH was 75 ppm, 10 ppm, and 0.1 mM, respectively. Because of low production at low overpotentials and the detection limit of GC and HPLC, experiments were performed three times to ensure the experimental reproducibility. Total FEs of all products with error bars are around 100% at all electrodes without further normalization.

### In Situ ATR-SEIRAS

A thin layer of poly Cu or Au (ca. 70 nm thickness) was deposited on either a silicon (for Cu) or ZnSe (for Au) prism via magnetron sputtering machine (Leybold, Z-400). The obtained Cu and Au films were used as working electrode during in situ ATR-SEIRAS experiments with an IR spectrometer (Bruker, Vertex 80v). To avoid H_2_O formation from HCO_3_^–^, 0.1 M KDCO_3_ solution was prepared by dissolving 0.05 M K_2_CO_3_ in D_2_O and subsequent saturation with CO_2_, which was then used as the working electrolyte in all experiments in D_2_O solutions. The background spectra were taken at open circuit potential in H_2_O or D_2_O (Sigma, 99.9 atom % D) before each experiment. Afterward, the working electrolyte was added to the cell. Before CO_2_ reduction experiments, cyclic voltammograms were taken in CO_2_ saturated solutions to initialize the surface until the spectra were stable. After that, the sample spectra were taken during LSV from 0.3 V vs RHE to target negative potentials on different working electrode at 1 mV s^–1^. All spectra were taken in absorbance mode by averaging over 200 scans with a 4 cm^–1^ resolution.

## Supporting Information Available

More data on AFM, voltammetry, in situ ATR-SEIRA spectra, and contact-angle measurements (PDF)
