# Supplementary material for: Enhanced Electrochemical CO2 Reduction to Formate on Poly(4-vinylpyridine)-Modified Copper and Gold Electrodes
Source: ACS Appl Mater Interfaces. 2022 Sep 27;14(40):45263–71. doi: 10.1021/acsami.2c10452 (PMC9562278; doi:10.1021/acsami.2c10452)
Supplement: Supplementary file 1 — am2c10452_si_001.pdf [file am2c10452_si_001.pdf]

Supporting Information

Enhanced Electrochemical CO_2 Reduction to Formate on Poly(4-vinylpyridine)-modified Copper and Gold Electrodes

*Chunmiao Ye, Stefan J. Raaijman, Xiaoting Chen and Marc T.M. Koper**

Leiden Institute of Chemistry, Leiden University, 2300 RA Leiden, The Netherlands

*email: m.koper@chem.leidenuniv.nl

Table of contents

1. **The morphology of our working electrodes before electrochemical experiments 3**
2. **Blank voltammetry and chronoamperometry experiments on unmodified and P4VP-modified pyrolytic graphite disk electrodes 4**
3. **Summary of vibrational bands assignments with different infrared spectroscopy 5**
4. **ATR-SEIRAS on poly Cu before adding P4VP layer 6**
5. **ATR-SEIRAS on poly Au and P4VP-modified Au during CO_2RR 7**
6. **Contact angle measurements on the working electrodes before electrochemical experiments 8**
7. **Cyclic Voltammetry on P4VP-modified Cu and Au electrodes after CO_2RR 9**

References 10

1. The morphology of working electrodes before electrochemical experiments

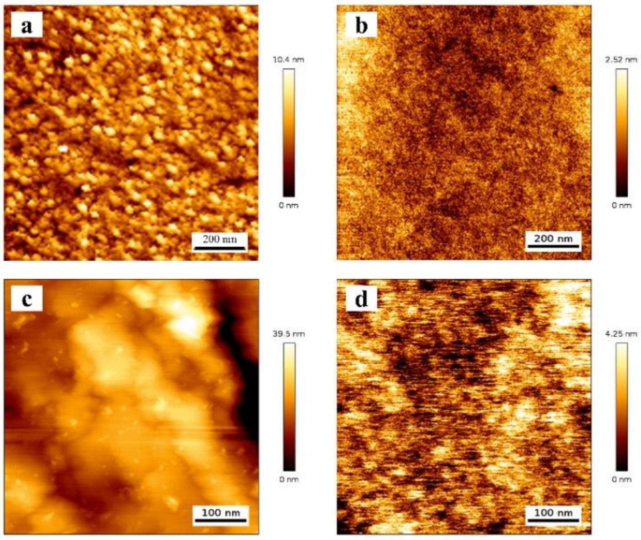

Figure S1. AFM images of (a) poly Cu, (b) P4VP-modified Cu, (c) poly Au and (d) P4VP-modified Au in the air before all experiments.

2. Blank voltammetry and chronoamperometry experiments on unmodified and P4VP-modified pyrolytic graphite disk electrodes

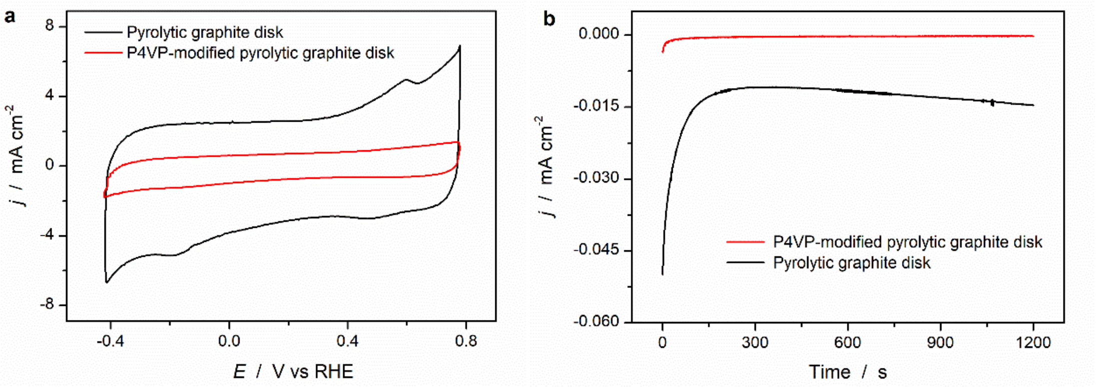

Figure S2. (a) Cyclic voltammograms with scan rate 50 mV/s, and (b) total current density of CO_2 reduction on P4VP modified pyrolytic graphite (red curve) and unmodified pyrolytic graphite electrodes (black curve) at -0.6 V vs RHE in CO_2 saturated 0.1 M $KHCO_3$ solution. With reversible hydrogen electrode as reference electrode. All potentials were IR corrected.

3. Summary of vibrational bands assignments with different infrared spectroscopy

Table S1 Vibrational Assignments of the ATR-SEIRAS bands

Surface	Band center / cm^{-1}	Species	Method	assignment
[1]	2343	solution CO_2	IR	ν_{COO}
Cu (110)[2-3]	2070	adsorbed CO on (100) or (111) step site	SNIFTIRS	
Cu (100)[4]	2050	adsorbed CO on (100) terrace	SNIFTIRS	
Cu single crystals,[5] poly Cu[6]	2001-1933	adsorbed CO on bridge site	SNIFTIRS	
[7]	1650	H_2O	IR	δ_{HOH}
poly Au[8] and poly Cu[9]	1620	solution HCO_3^-	ATR-SEIRAS	$\nu_{COO,\ asym}$
poly Cu[9]	1616-1608	adsorbed $HCOO^-$	ATR-SEIRAS	$\nu_{COO,\ asym}$
poly Cu,[9] Cu(100),[4] Au(111)[10]	1533-1508	adsorbed CO_3^{2-}	FTIR, IRAS	$\nu_{COO,\ asym}$
Poly Cu,[9] Au(111)[10] and poly Au[11]	1410	solution CO_3^{2-}	ATR	$\nu_{COO,\ asym}$
	1365	solution HCO_3^-	ATR	$\nu_{COO,\ sym}$
	1330	solution HCO_3^-	ATR-SEIRAS	

4. ATR-SEIRAS on poly Cu before adding P4VP layer

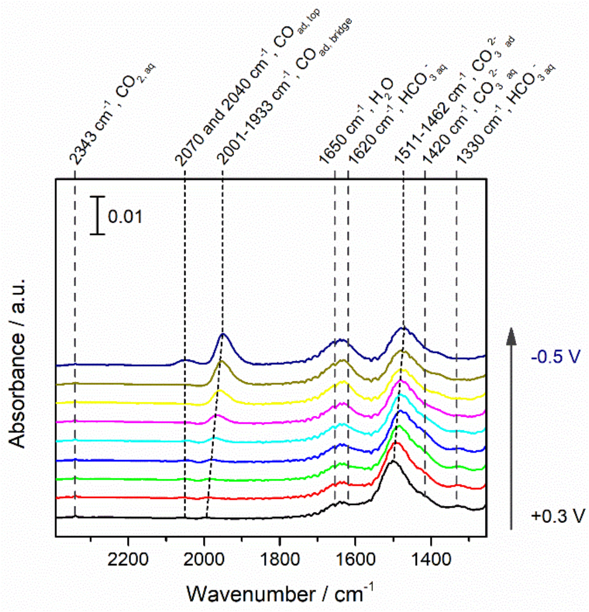

Figure S3. ATR-SEIRA spectra of CO_2RR on poly Cu during linear sweep voltammetry at 1 mV/s from 0.3 V to -0.5 V vs RHE in CO_2 saturated 0.1M $KHCO_3$ aqueous solution. The background spectrum was taken at OCP in H_2O before experiments. The potential interval between spectra is 0.1 V.

5. ATR-SEIRAS on poly Au and P4VP-modified Au during CO_2RR

The ratio of the integrals of the bands related to dissolved CO_2 and solution HCO_3^- (2343 and 1620 cm^{-1}, respectively; $I_{CO_2} / I_{HCO_3^-}$) was calculated as an indicator of local environment. To avoid the influence of H_2O bending band at 1650 cm^{-1}, D_2O was used to prepare the electrolyte. Bands related with dissolved CO_2, solution HCO_3^-, and solution CO_3^{2-} on unmodified and P4VP-modified Au electrodes are shown in Figures S4 a and b respectively. Additionally, the bands related with solution HCO_3^- and solution CO_3^{2-} (at 1360 and 1460 cm^{-1}) show the same information of the different local environment changes on unmodified and P4VP-modified Au electrodes. When the potential went to -0.6 V vs RHE, the intensity of the band related with solution HCO_3^- decreases, while the intensity of the band related with solution CO_3^{2-} increases (compared with the bands intensity at 0.3 V vs RHE). Due to the growth of the broad band related with solution CO_3^{2-}, bands at 1360 and 1460 cm^{-1} overlap, which slightly shifts the band center of solution CO_3^{2-}. However, this observation does not have any effect on bands assignment nor on the calculated value of $I_{CO_2} / I_{HCO_3^-}$.

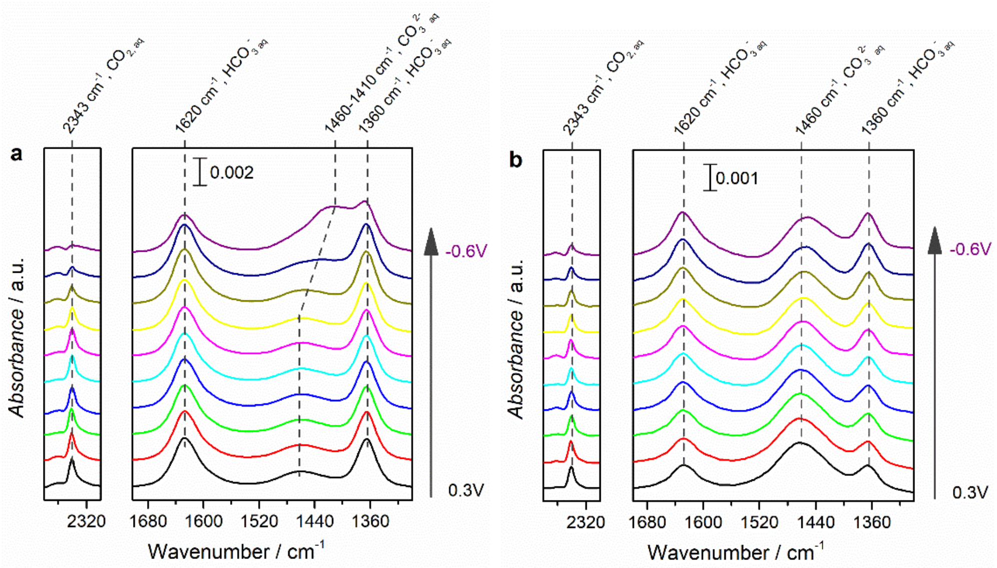

Figure S4. ATR-SEIRA spectra of CO_2RR on the poly Au (a) and P4VP-modified Au (b) during linear sweep voltammetry at 1 mV/s from 0.3V to -0.6V vs RHE in CO_2 saturated 0.05M K_2CO_3 dissolved in D_2O. The background spectrum was taken at OCP in D_2O before experiments. The potential interval between spectra is 0.1 V.

6. Contact angle measurements on the working electrodes before electrochemical experiments

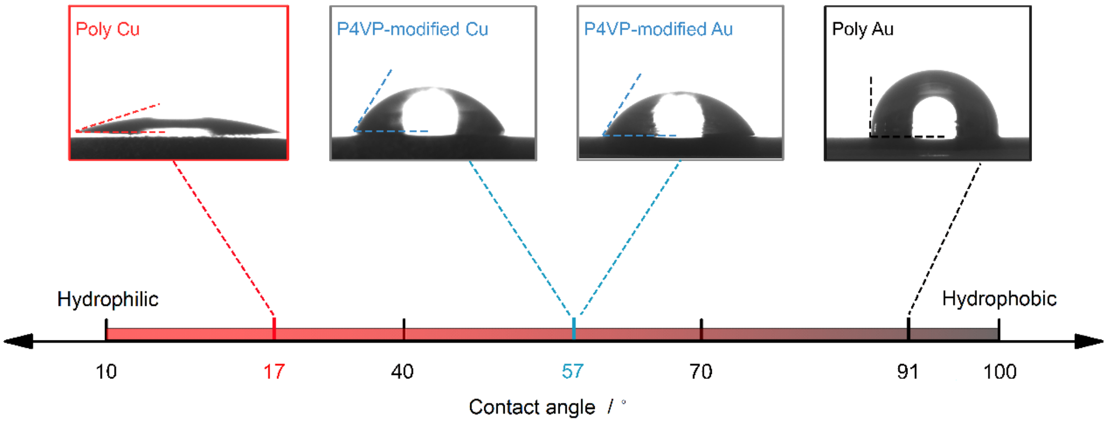

Figure S5. Water contact angles on poly Cu, P4VP-modified Cu, poly Au and P4VP-modified Au surfaces.

7. Cyclic Voltammetry on P4VP-modified Cu and Au electrodes after CO_2RR

Cyclic Voltammetry was employed to characterize the electrodes after CO_2RR experiments. Although CO oxidation peak was observed on P4VP-modified Au electrode after CO_2RR at -0.5 V vs RHE,[12] the cyclic voltammograms on both P4VP-modified Cu and Au electrodes exhibit minor changes after applying negative potentials, as shown in the following cyclic voltammograms of P4VP-modified Cu and Au electrodes. This result indicates the intactness of the P4VP layer on poly Cu and Au surfaces after CO_2RR.

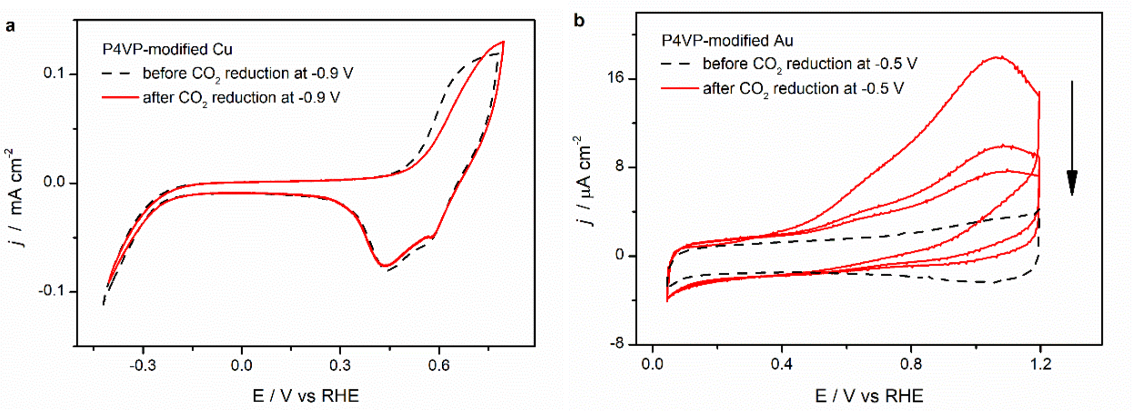

Figure S6. Cyclic voltammograms of (a) P4VP-modified Cu after CO_2RR at -0.9 V vs RHE and (b) P4VP-modified Au after CO_2RR at -0.5 V vs RHE, measured at 50 mV/s in CO_2 saturated 0.1 M $KHCO_3$ solution.

REFERENCE

(1) Falk, M.; Miller, A. G. Infrared Spectrum of Carbon Dioxide in Aqueous Solution. *Vib. Spectrosc* **1992,** *4*, 105-108.

(2) Koga, O.; Teruya, S.; Matsuda, K.; Minami, M.; Hoshi, N.; Hori, Y. Infrared Spectroscopic and Voltammetric Study of Adsorbed CO on Stepped Surfaces of Copper Monocrystalline Electrodes. *Electrochim. Acta* **2005,** *50*, 2475-2485.

(3) Gunathunge, C. M.; Li, X.; Li, J.; Hicks, R. P.; Ovalle, V. J.; Waegele, M. M. Spectroscopic Observation of Reversible Surface Reconstruction of Copper Electrodes under CO_2 Reduction. *J. Phys. Chem. C* **2017,** *121*, 12337-12344.

(4) Hori, Y.; Koga, O.; Watanabe, Y.; Matsuo, T. Ftir Measurements of Charge Displacement Adsorption of CO, on Poly- and Single Crystal (100) of Cu Electrodes. *Electrochim. Acta* **1998,** *44*, 1389-1395.

(5) Shaw, S. K.; Berna, A.; Feliu, J. M.; Nichols, R. J.; Jacob, T.; Schiffrin, D. J. Role of Axially Coordinated Surface Sites for Electrochemically Controlled Carbon Monoxide Adsorption on Single Crystal Copper Electrodes. *Phys. Chem. Chem. Phys.* **2011,** *13*, 5242-5251.

(6) Gunathunge, C. M.; Ovalle, V. J.; Li, Y.; Janik, M. J.; Waegele, M. M. Existence of an Electrochemically Inert CO Population on Cu Electrodes in Alkaline Ph. *ACS Catal.* **2018,** *8*, 7507-7516.

(7) Larsen, O. F.; Woutersen, S. Vibrational Relaxation of the H_2o Bending Mode in Liquid Water. *J. Chem. Phys.* **2004,** *121*, 12143-12145.

(8) Ayemoba, O.; Cuesta, A. Spectroscopic Evidence of Size-Dependent Buffering of Interfacial Ph by Cation Hydrolysis During CO_2 Electroreduction. *ACS Appl. Mater. Interfaces* **2017,** *9*, 27377-27382.

(9) Moradzaman, M.; Mul, G. Infrared Analysis of Interfacial Phenomena During Electrochemical Reduction of CO_2 over Polycrystalline Copper Electrodes. *ACS Catal.* **2020,** *10,* 8049-8057.

(10) Arihara, K.; Kitamura, F.; Ohsaka, T.; Tokuda, K. Characterization of the Adsorption State of Carbonate Ions at the Au(111) Electrode Surface Using in Situ Iras. *J. Electroanal. Chem.* **2001,** *510*, 128-135.

(11) Dunwell, M.; Yang, X.; Setzler, B. P.; Anibal, J.; Yan, Y.; Xu, B. Examination of near-Electrode Concentration Gradients and Kinetic Impacts on the Electrochemical Reduction of CO_2 Using Surface-Enhanced Infrared Spectroscopy. *ACS Catal.* **2018,** *8,* 3999-4008.

(12) Marcandalli, G.; Villalba, M.; Koper, M. T. M. The Importance of Acid-Base Equilibria in Bicarbonate Electrolytes for CO_2 Electrochemical Reduction and CO Reoxidation Studied on Au(Hkl) Electrodes. *Langmuir* **2021,** *37*, 5707-5716.
